# Supplementary figures and images for: Oral bacteria induce IgA autoantibodies against a mesangial protein in IgA nephropathy model mice
Source: Life Sci Alliance. 2024 Feb 8;7(4):e202402588. doi: 10.26508/lsa.202402588 (PMC10853438; doi:10.26508/lsa.202402588)

Figure 1

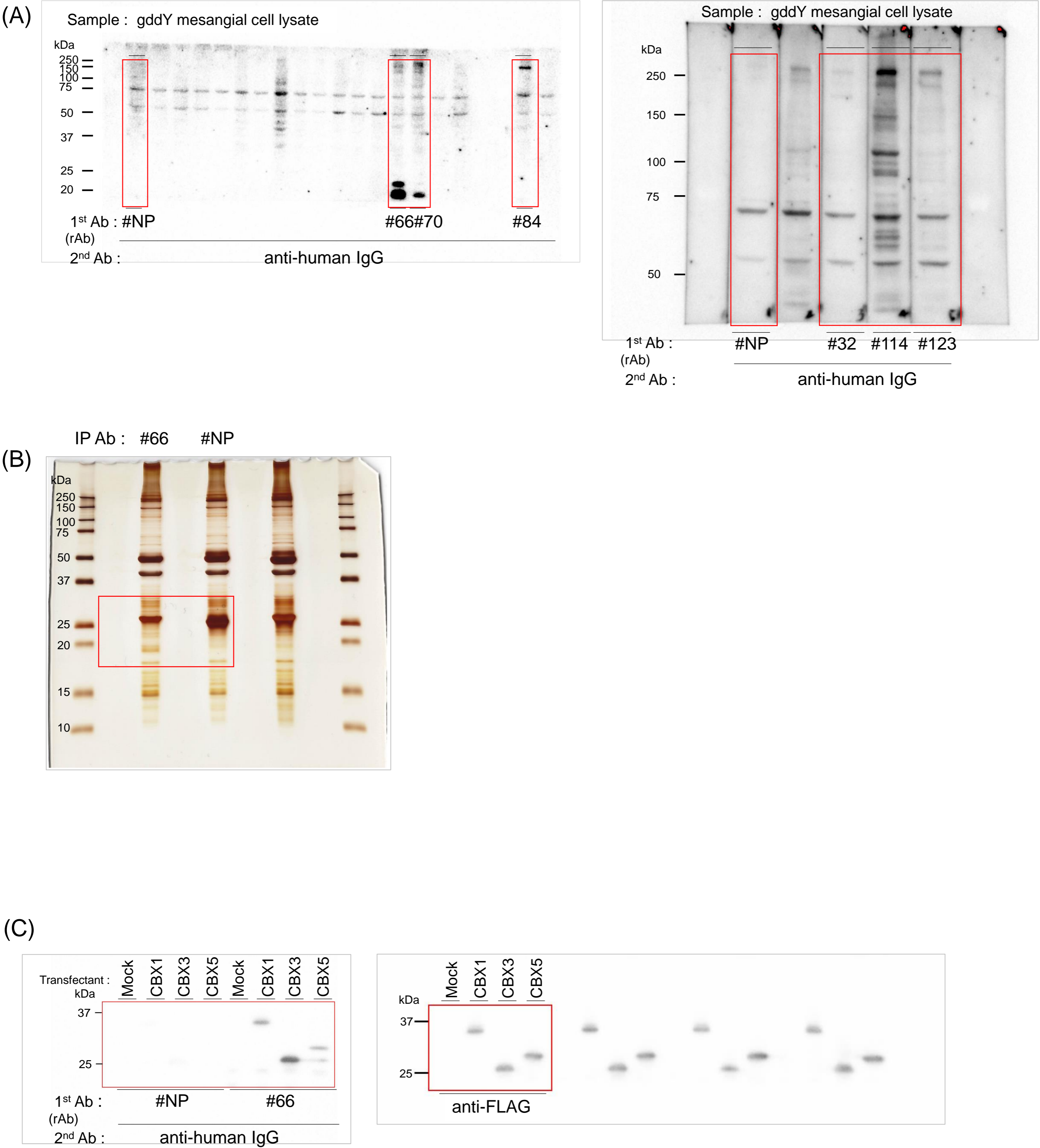

(E)

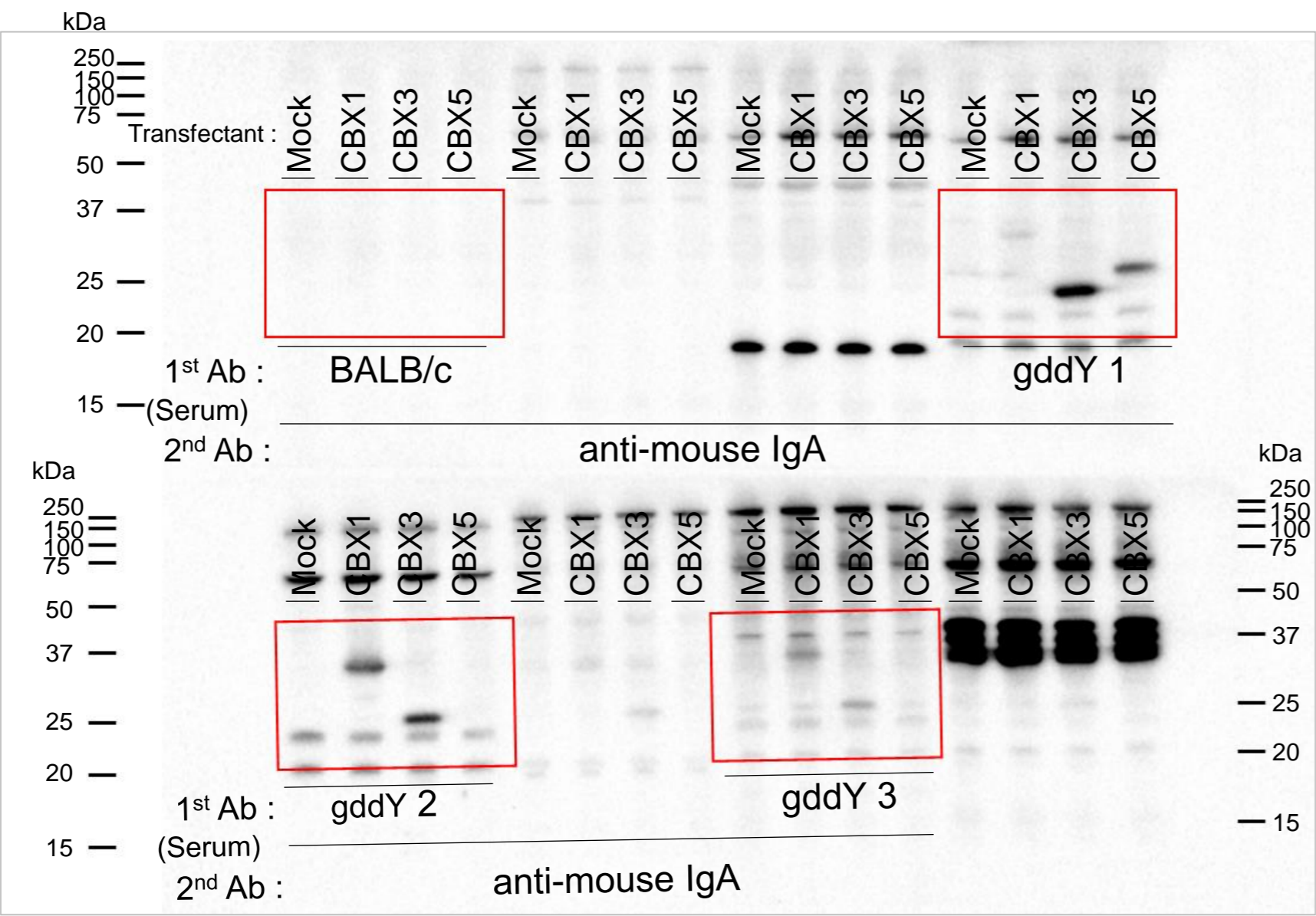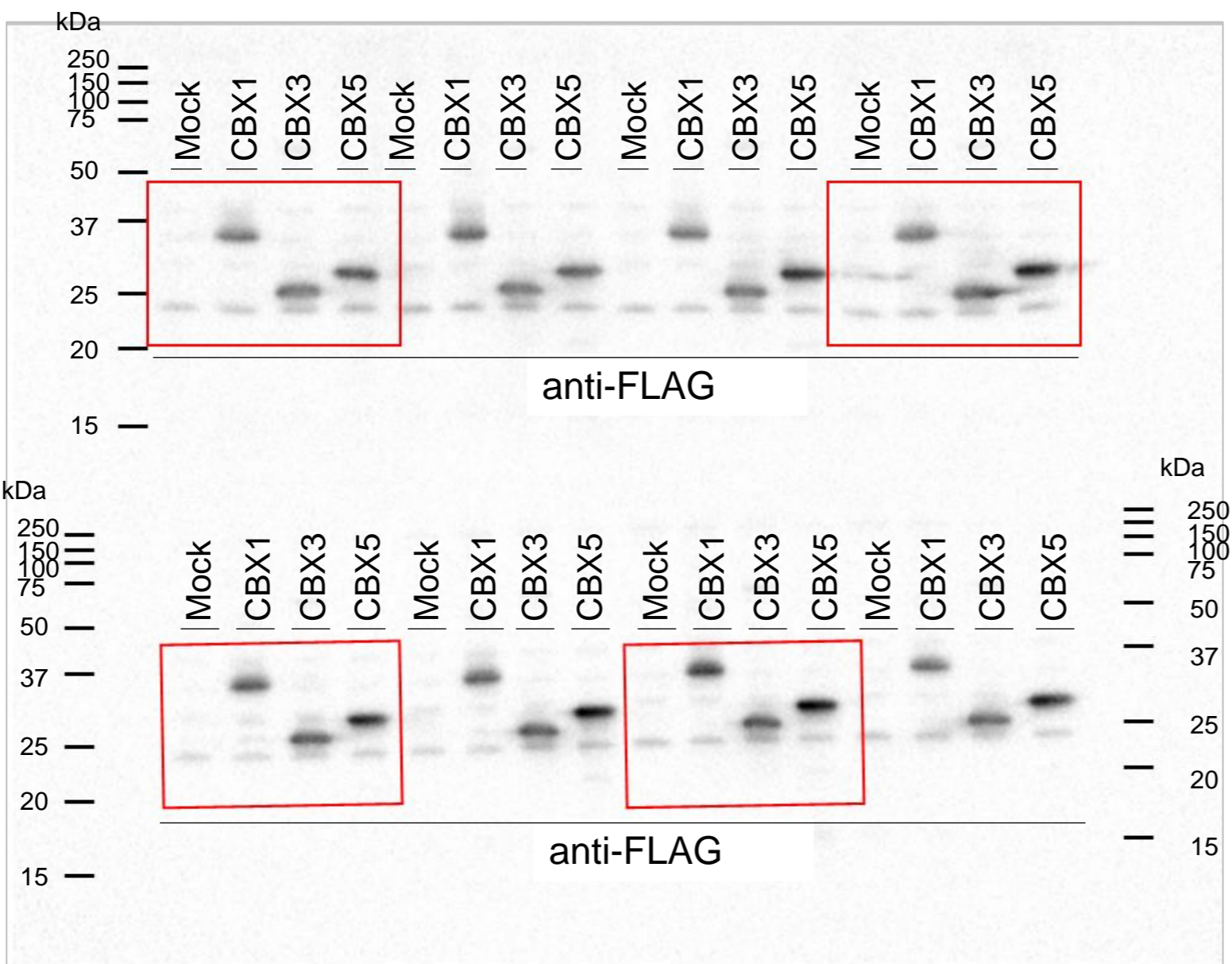

Supplement: Supplementary file 1 [file LSA-2024-02588_SdataF1.pdf]

Figure S1

(D)

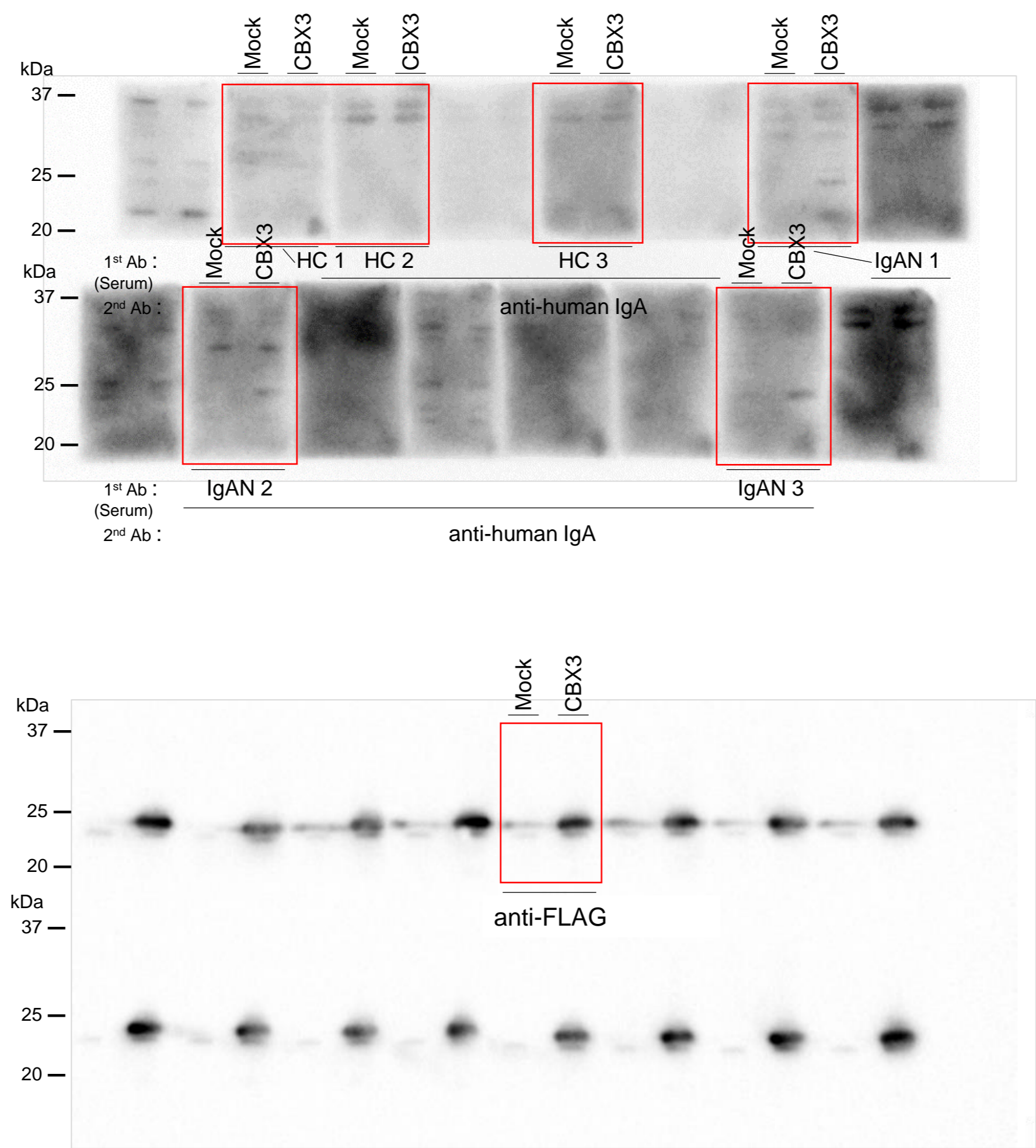

Supplement: Supplementary file 2 [file LSA-2024-02588_SdataFS1.pdf]

Figure 2

(D)

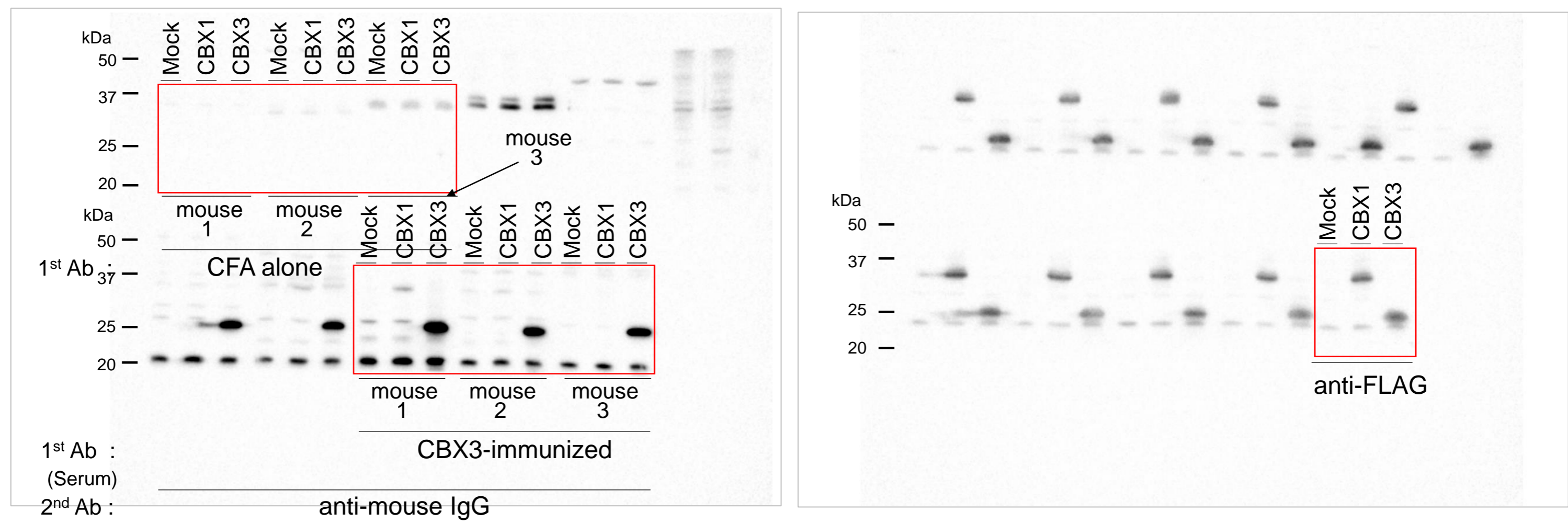

Supplement: Supplementary file 3 [file LSA-2024-02588_SdataF2.pdf]

Figure S2

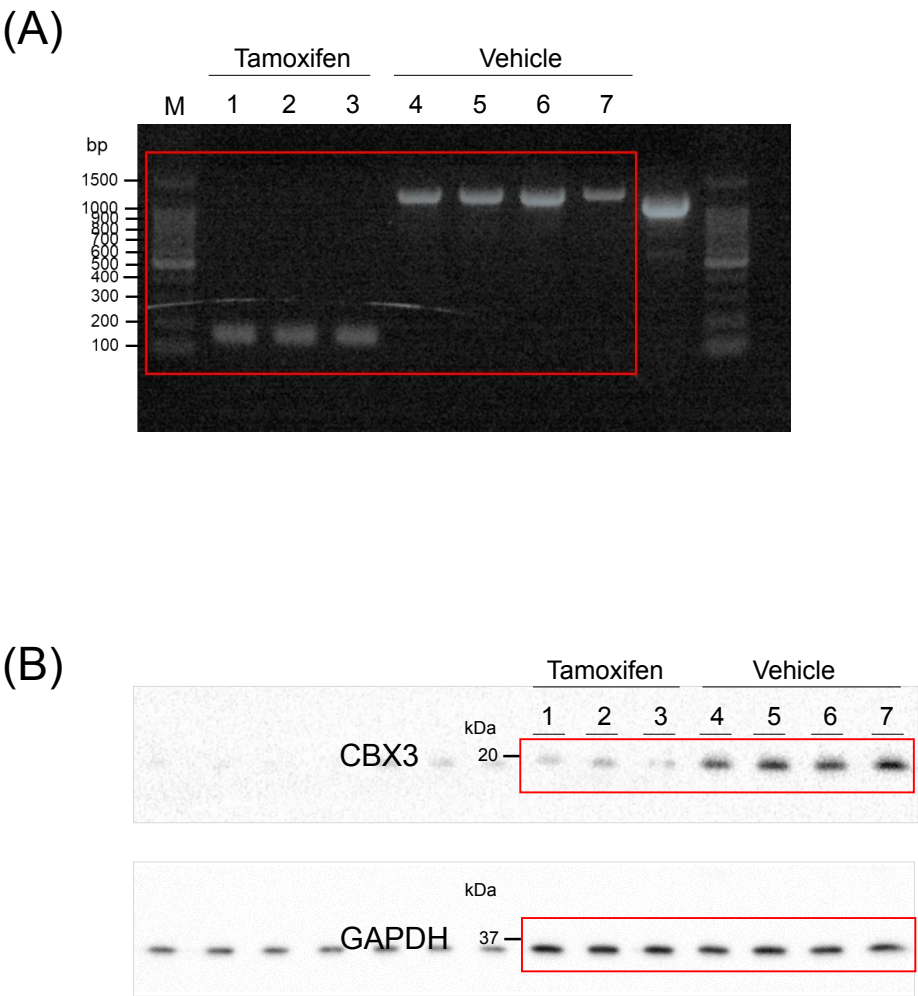

Supplement: Supplementary file 4 [file LSA-2024-02588_SdataFS2.pdf]

Figure 3

(E)

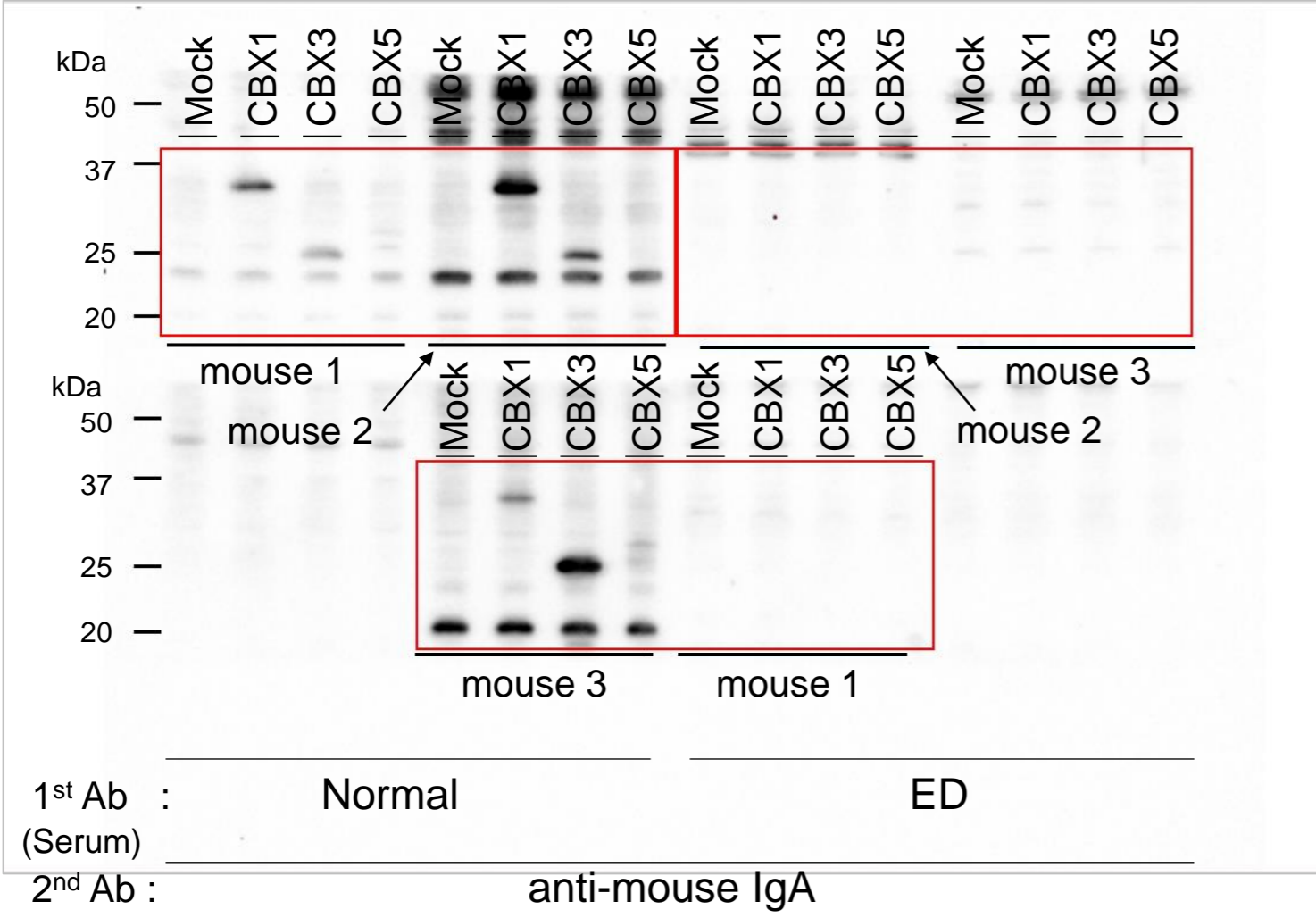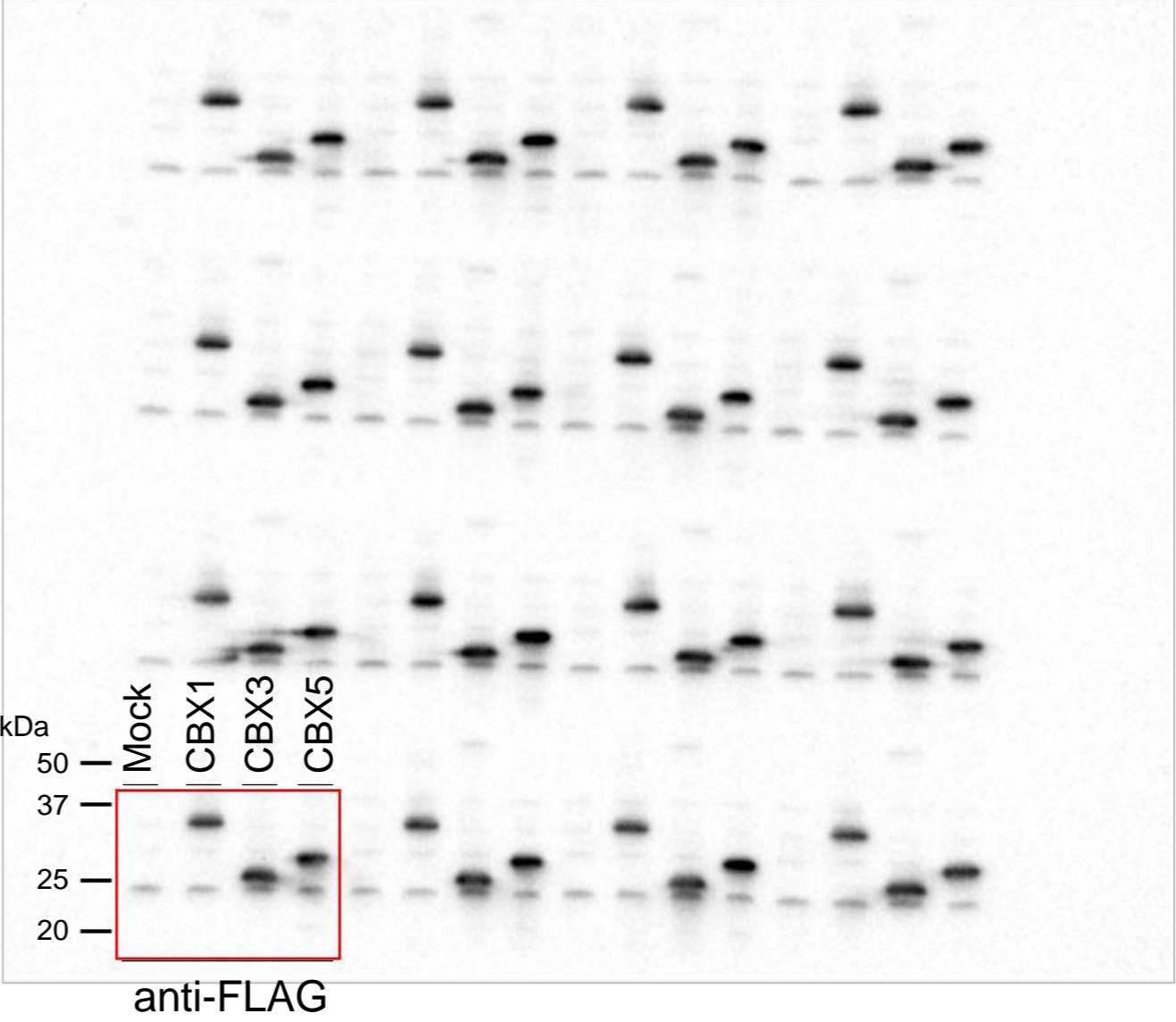

Supplement: Supplementary file 5 [file LSA-2024-02588_SdataF3.pdf]

Figure 4

(C)

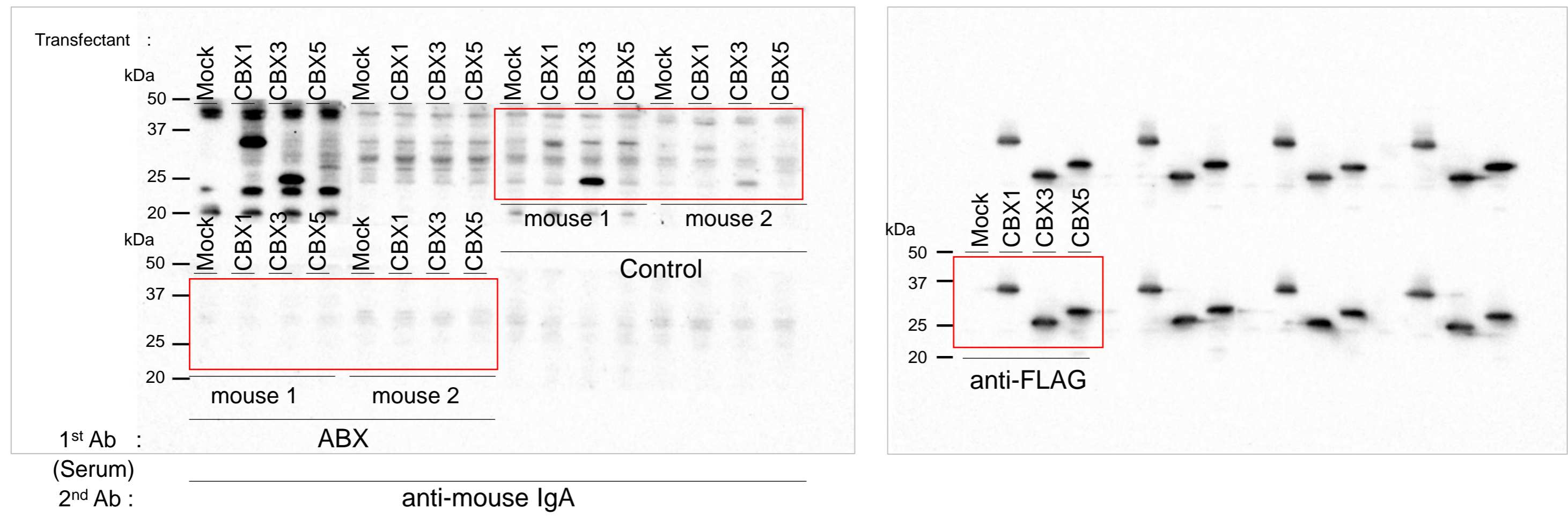

Supplement: Supplementary file 6 [file LSA-2024-02588_SdataF4.pdf]

Figure 5

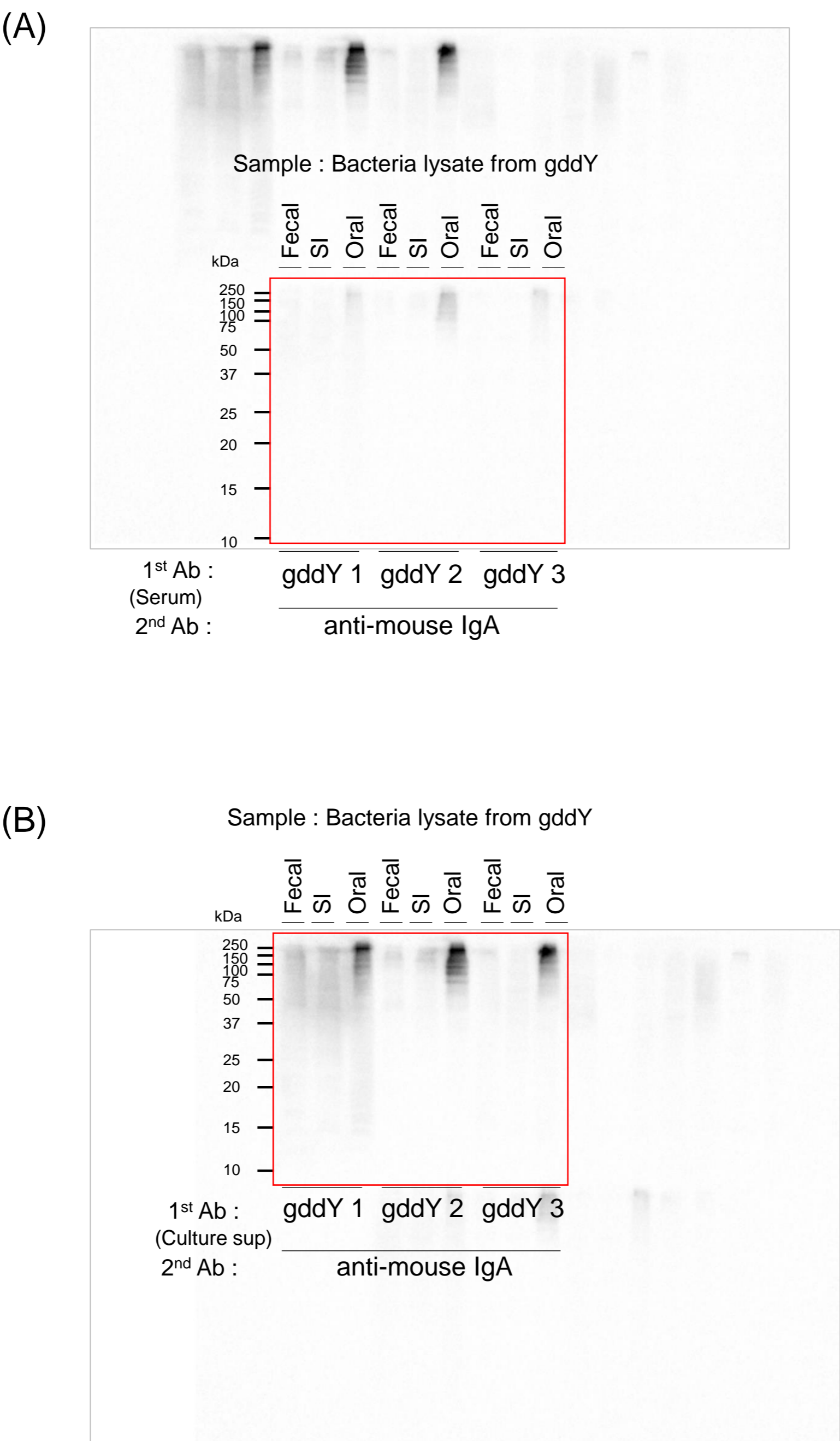

Supplement: Supplementary file 7 [file LSA-2024-02588_SdataF5.pdf]

Figure S4

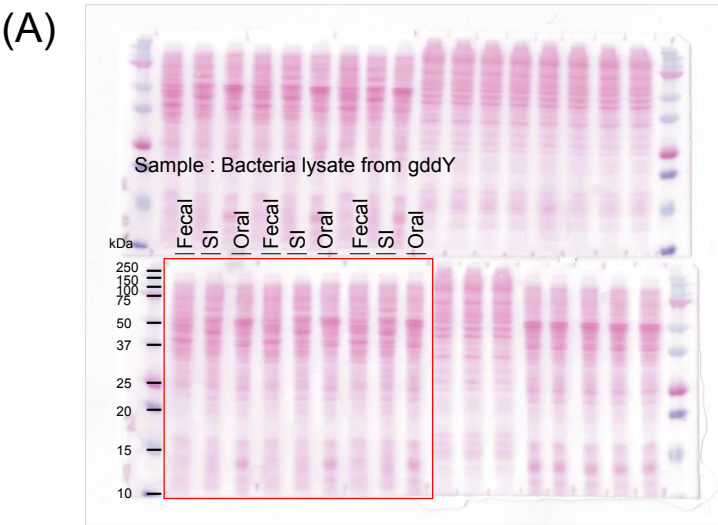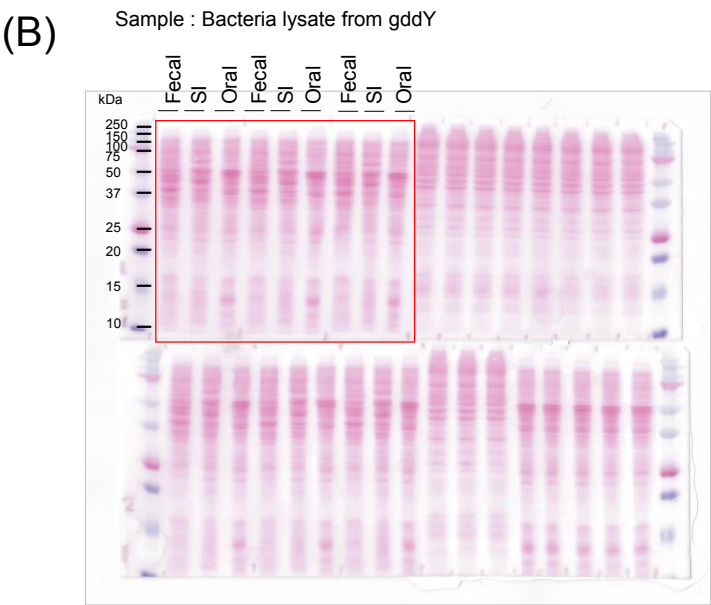

Supplement: Supplementary file 8 [file LSA-2024-02588_SdataFS4.pdf]

Figure 6

(D)

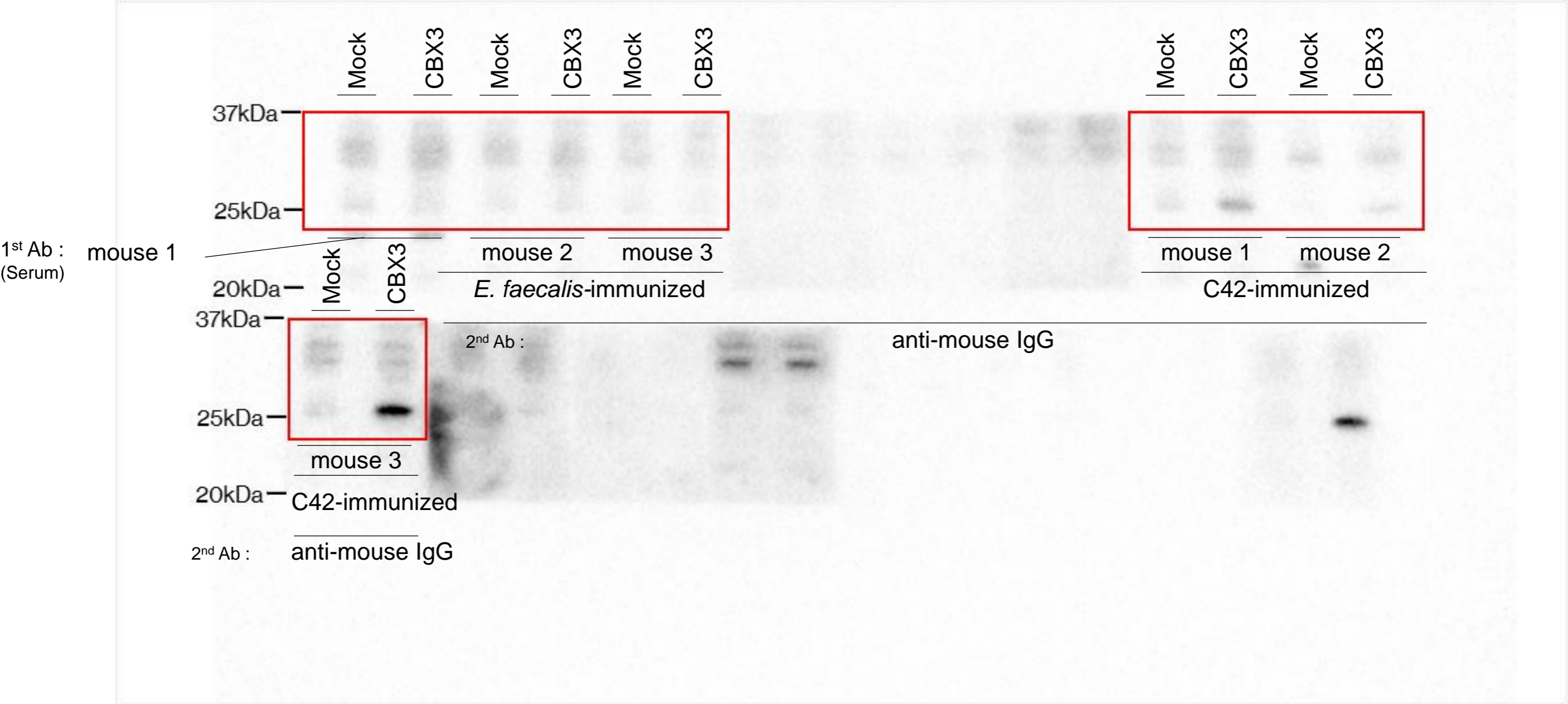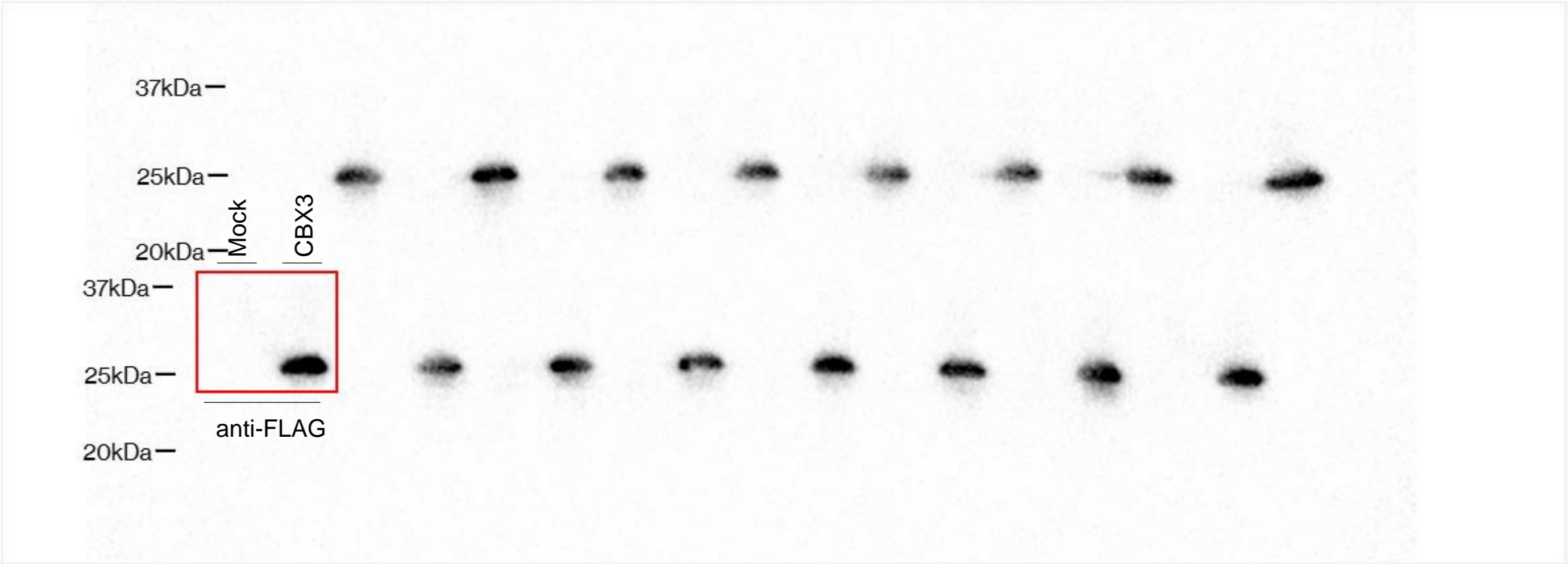

Supplement: Supplementary file 9 [file LSA-2024-02588_SdataF6.pdf]

Figure 7

(B)

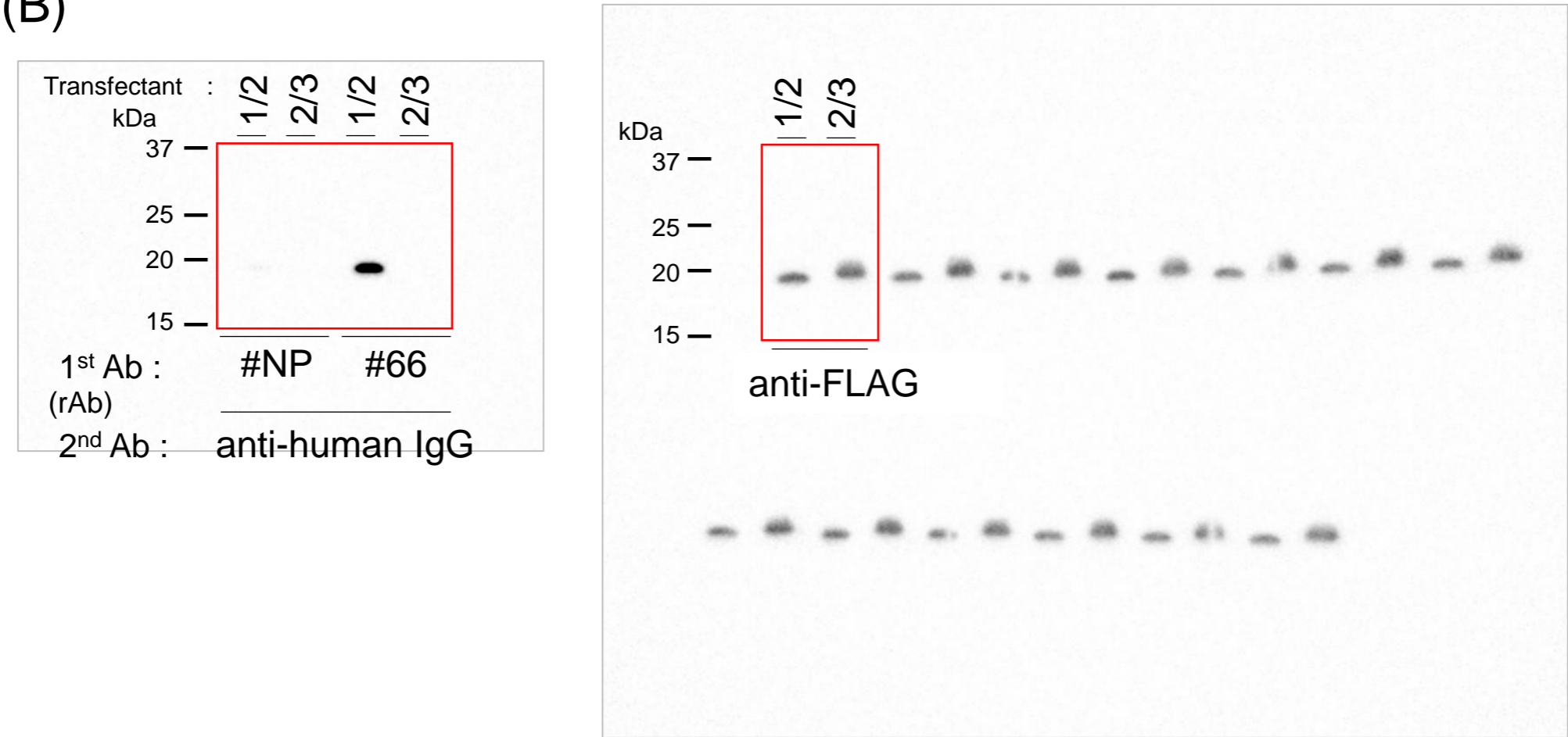

(C)

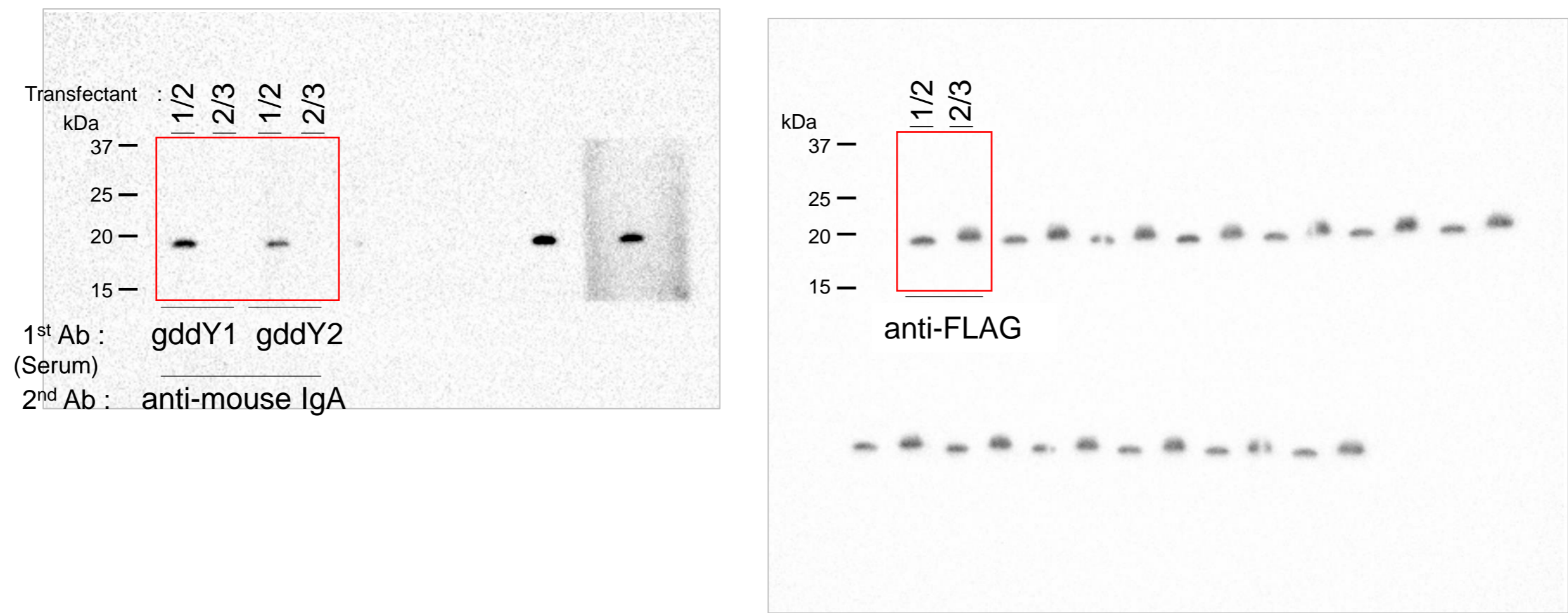

(D)

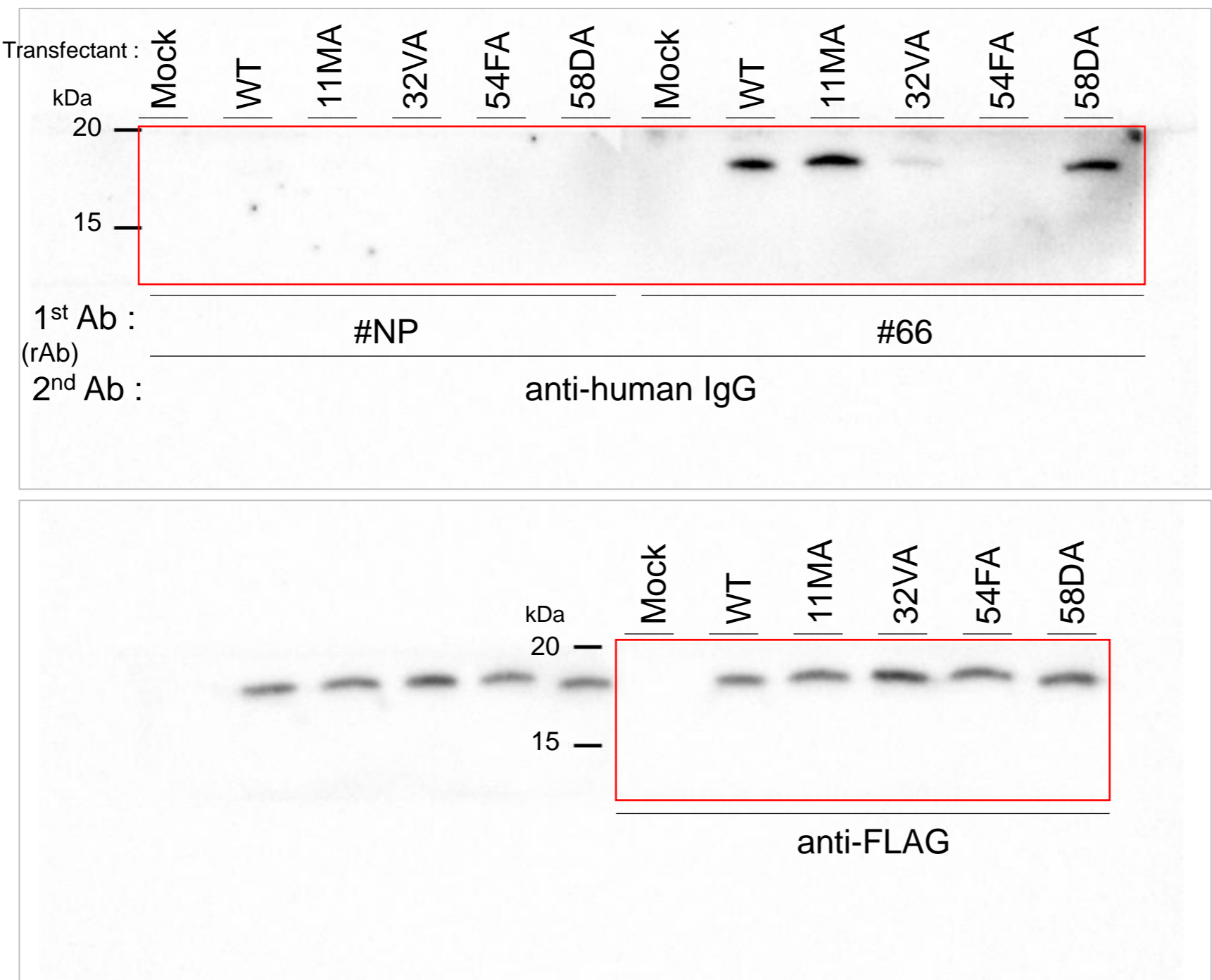

Supplement: Supplementary file 10 [file LSA-2024-02588_SdataF7.pdf]
